# Retrospective evaluation of radiofrequency volumetric tissue reduction for hypertrophic turbinates in dogs with brachycephalic obstructive airway syndrome

**DOI:** 10.1371/journal.pone.0306391

**Published:** 2024-07-01

**Authors:** Marie-Cécile von Doernberg, Brigitte von Rechenberg, Henning Richter

**Affiliations:** 1 HNO-Chirurgie von Doernberg, Alzey, Germany; 2 Center for Applied Biotechnology and Molecular Medicine (CABMM), University of Zurich, Zurich, Switzerland; 3 Diagnostic Imaging Research Unit (DIRU), Clinic for Diagnostic Imaging, Vetsuisse Faculty, University of Zurich, Zurich, Switzerland; University of Camerino, ITALY

## Abstract

**Objective:**

The objective of this study was to retrospectively assess the effect of Radiofrequency Volumetric Tissue Reduction (RFVTR) on hypertrophic turbinates and clinical outcome in brachycephalic dogs when included in multi-level surgery (MLS).

**Study design:**

Clinical retrospective multicenter study.

**Animals:**

132 client-owned brachycephalic dogs.

**Methods:**

132 brachycephalic dogs with high-grade Brachycephalic Obstructive Airway Ayndrome (BOAS) and hypertrophic turbinates were treated with RFVTR as part of MLS of the upper airways. Intranasal obstruction was evaluated by computer tomography (CT) and antero-/retrograde rhinoscopy before and 6 months after RFVTR. The clinical records, the CT images and the rhinoscopy videos were reviewed and clinical evolution was evaluated using a standardized questionnaire. The data was scored semi-quantitatively.

**Results:**

In this study, 132 patients were included for a follow-up period of 120 weeks. RFVTR resulted in minor complications, including serous nasal discharge within the first postoperative week in all dogs, and intermittent nasal congestion between 3–8 weeks after treatment in 24.3% of the patients. Rhinoscopy and CT follow-ups were available for 33 patients. Six months after treatment intranasal airspace was increased (p = 0.002) and the presence and overall amount of mucosal contact points was reduced (p = 0.039).

**Conclusion:**

MLS with RFVTR led to a significant reduction in turbinate volume at the 6-month follow-up examination and significant clinical improvement over a long-term period of 120 weeks. This suggests the viability of RFVTR as a turbinate-preserving treatment for intranasal obstruction in dogs with BOAS.

**Clinical significance:**

RFVTR is a minimally invasive turbinoplasty technique for intranasal obstruction in dogs with BOAS and can be included in MLS without increasing complication rates.

## Introduction

Brachycephalic Obstructive Airway Syndrome (BOAS) is caused by shortening of the facial skull and subsequent narrowing of the upper airways [1]. To date the main components of BOAS include stenotic nares, hypertrophic or aberrant nasal turbinates, dynamic pharyngeal collapse, elongated and thickened soft palate, laryngeal collapse, and hypoplastic trachea [2–6]. Fernández-Parra et al., 2019, showed that upper airway resistance and upper airway pressure were 9.6 and 8.4 times higher in brachycephalic breeds than in mesocephalic or dolichocephalic breeds, respectively [7]. Traditional multilevel surgery (MLS), addressing stenotic nares, elongated soft palate, and resection of everted sacculi alone, produces variable results and does not seem to alleviate upper airway obstruction enough [8–10]. Since Oechtering et al. published their work on aberrant turbinates a more in-depth understanding of intranasal obstruction in BOAS has led to a broader application of complex MLS, including resection of the turbinates that were considered to cause intranasal obstruction [5, 11, 12]. The Laser-assisted-Turbinectomy (LATE) is a turbinate reduction procedure. With the aid of a diode-laser the maxilloturbinates and/or parts of the ethmoturbinates are removed to increase nasal airspace, which in theory would lead to improved air- passage, and less breathing effort [13, 14]. The effect of turbinectomy on nasal airflow and cooling efficiency has not been studied in dogs, so far. In human patients, airflow patterns before turbinectomy were more evenly distributed than post-operatively. The lack of turbinates created a narrow stream of air that travelled with increased velocity to the nasopharynx without interacting with the remaining mucosa, causing a reduction in air-conditioning (warming, humidification and filtration) [15, 16]. Furthermore, prolonged hospitalization, potentially life-threatening hemorrhage, post-operative death, scarring, synechia-formation, secondary atrophic rhinitis (dry nose syndrome), and rapid turbinate regrowth have been described as early or late complications after turbinectomy in humans [17–19]. One publication on 158 dogs undergoing LATE also describes major postoperative complications, including rapid turbinate regrowth at 6-month follow-up in 15.8%, and synechia formation in 3% [12]. While hospitalization after MLS including LATE has been described as standard protocol, post-operative hospitalization is recognized as a risk factor in brachycephalic breeds [12, 20].

The physiology of respiration and olfaction in the dog differs from humans, who are microsmats in contrast to dogs, which qualify as macrosmats. All macrosmats have a destined area in the nose for olfaction called the respiratory recess, or respiratory cleft [21–25]. During normal breathing the inhaled air is mixed in the nasal vestibule and divided into the respiratory and the olfactory pathway. The olfactory pathway is unidirectional and runs along the dorsal meatus towards the respiratory recess, which is situated in the dorsal back of the nose and divided from the respiratory pathway by the vomer, or lamina transversa [21–23]. The olfactory recess is occupied by ethmoturbinates covered in olfactory epithelium [26]. The respiratory pathway leads the air through the labyrinth of the maxilloturbinates, the common and the ventral meatuses, and the nasopharynx and larynx towards the lower airways. The outbreath bypasses the olfactory recess but mandatorily travels through the maxilloturbinates where the moisture of the air is regained [27]. The maxilloturbinates and the rostral part of the first ethmoturbinate are covered in ciliated, respiratory epithelium [26–29]. In maxilloturbinates, unlike in ethmoturbinates, the lamina propria is augmented with a rich venous plexus, regulated by the autonomous nervous system. Fluctuations in venous diameter cause periodic change in mucosal thickness: the nasal cycle, which is a phenomenon dogs share with humans [30–32]. Engorgement of the turbinates is triggered by an increase in parasympathetic tone, cold air, and hypocapnia. Increased sympathetic tone, exercise, and hypercapnia lead to a reduction in engorgement [33]. Dogs rely on hyperventilation of their upper airways to regulate core body temperature [27]. Turbinectomy in dogs may not only cause flow-dynamic changes but may also have a detrimental effect on cooling-ability by decreasing the overall amount of venous plexus and mucosal surface area. Rapid regrowth after LATE has been stated as a positive development, based on the interpretation that regrowth of resected turbinals occurred in a healthy manner, but studies showing long-term follow-up after LATE, and studies on fluid-dynamics, histology, and mucosal function testing after LATE are not available to date [34].

In human medicine treatment of hypertrophic turbinates has been researched intensely for over 120 years [19, 35, 36]. More than three decades ago, increased knowledge of the importance of turbinate function coupled with technological advances have led to a drastic decline in the number of turbinectomies in favor of turbinoplasty surgery [37–39]. RFTVR is routinely performed under local anesthesia as an outpatient procedure [39–48]. Radiofrequency Ablation (RFA) is a subset of radiofrequency electrosurgery. RFA uses high-frequency alternating current with an electromagnetic spectrum between 300 and 500 kHz. The difference between RFA and RF electrosurgery lies in the way the alternating current is transferred to the tissue and the tissue effects. Protein denaturation and desiccation occur at an exposure to 60°C—95°C. With 100°C the water in the cells boils causing explosive evaporation. Above 200°C the tissue is broken down into its organic molecules, evident as a blackening or charring called carbonization [49, 50]. RFA devices were developed for protein coagulation only, by limiting the temperature range to 40–95°C [51]. The effect on the tissue depends on exposure time, tissue-impendence and the setting of the power [49]. In RFA an active electrode is placed inside the tissue. The electromagnetic energy of the alternating current is converted into kinetic energy at the level of the intracellular ions and then into thermal energy. The thermal energy breaks protein bonds causing protein denaturation. Heat conduction to deeper layers is minimal due to the low temperature range [19, 52–54]. RFA has been used in a wide range of applications in veterinary medicine, including ligament laxity, the soft palate, the vocal folds, minimal invasive cardiac surgery, and tumor reduction procedures [55–62]. With a bipolar probe the RF current travels between electrodes and does not pass through the patient. Ablation zones around bipolar probes are smaller than around monopolar probes [47, 49]. For the treatment of hypertrophic turbinates, the probe is inserted in the lamina propria between the mucous membrane and the bone. The distance between bone and mucosa in dogs is smaller compared to humans but will still safely accommodate a 1-mm diameter pediatric probe in most, making turbinoplasty a feasible therapy alternative to turbinectomy.

The objective of this study was to assess the effect of RFVTR on hypertrophic turbinates in dogs with BOAS, which were undergoing MLS. Additionally, we examined the clinical outcome over a period of up to 120 weeks. We hypothesized that RFTVR would be a feasible, efficient, and safe procedure to treat nasal congestion caused by hypertrophic turbinates in brachycephalic dogs and that due to its minimal invasive nature postoperative hospitalization could be avoided.

## Materials and methods

### Ethics statement

This is a retrospective multicenter analysis of a digital set of data, acquired from regular clinical patients during diagnostic and therapeutic work-up and follow-up. Standardized questionnaires, which were an integrative part of clinical care, were used to analyze follow-up information on the clinical outcome. Before evaluation the data was de-identified. Compared with standard of care clinical treatment, there was no additional stress, pain, or any kind of suffering for the animals, while using data for this study purpose.

### Study design

This retrospective study is based on a total of 132 cases of dogs affected with BOAS, which were treated with RFVTR as part of MLS in four different hospitals between January 2017 and June 2019. RFVTR was performed in all dogs with hypertrophic turbinates. Dogs without hypertrophic turbinates that underwent MLS were not included. The examinations and surgeries were performed by one board-certified surgeon (MCvD) according to a clinical standard protocol implemented in 2015. This clinical standard protocol also includes the video recording of the rhinoscopy, a full CT scan of the head, a re-evaluation after six months, and a follow-up obtained by phone or e-mail using a standardized questionnaire. Inclusion criteria for clinical cases consisted of a complete clinical history and examination, at least one high-quality CT dataset, one full rhinoscopy (anterograde and retrograde), MLS including RFVTR, and a minimum of 4 weeks follow-up period. The clinical records, rhinoscopy findings, and CT images were retrospectively assessed and assigned to semi-quantitative scores.

### Preoperative BOAS grading

The pre-operative examination consisted of a complete patient history and a clinical examination. The analyzed data originates from four veterinary clinics. For each patient all examinations (first and follow-up) were carried out in the same clinic. The history focused on the dog’s ability to exercise under different ambient temperatures, the duration of recovery after exercise, presence of sleep apnea, frequency of cyanotic episodes or collapse, and noisy breathing. The severity of BOAS was graded according to the Respiratory Function Grading Scheme (RFG scheme) from 0 = unimpaired to 3 = severely affected, based on the patient’s history and the clinical findings [2]. Briefly grade 0 (no BOAS) was defined as no respiratory noise present and no exercise intolerance. Grade 1 was defined as no respiratory noise at rest, intermittent noise audible without stethoscope during or after exercise. Grade 2 was defined as intermittent noise during exercise audible without auscultation and exercise impairment during warm ambient temperatures (>19°C). Grade 3 was defined as constant noise at rest, exercise impairment present at any ambient temperature, and/or prior cyanosis or syncope reported in the record. Prolonged recovery was defined as panting after exercise for any period longer than 5 minutes.

The body condition of the dogs was scored using four grades (grade 0 representing no overweight, grade 1 slight overweight, grade 2 moderate overweight, and grade 3 severe overweight). All dogs were premedicated with dexmedetomidine (0.003 g/kg) and butorphanol (0.2 mg/kg, IM). The dogs remained in a quiet room together with their owners until a deep plane of sedation was evident. Anesthesia was induced with propofol (1-3mg/kg, IV). Before transoral intubation, the presence and grade of the laryngeal collapse were assessed as described before [63]. General anesthesia was maintained with isoflurane (1.5–2%) in oxygen. All dogs received metoclopramide (0.5 mg/kg), ranitidine (2mg/kg), diazepam (0.2 mg/kg), amoxicillin clavulanic acid (12.5 mg/kg), dexamethasone (1-2mg/kg), and maropitant citrate (1mg/kg). Butorphanol administration was repeated every two hours until recovery. Local anesthesia was performed using a bilateral maxillary block (1mg/kg, Lidocaine, 2%). CT examination was performed in sternal recumbency with the upper jaw attached to a fixation device to allow the lower jaw and the tongue to drop. The hard palate was positioned parallel to the table, and perpendicular to the direction of the scan. The CT field of view ranged from the most rostral point of the lips to the external occipital protuberance. The clinics were equipped with individual CT scanners. The control CT was always performed at the same hospital where the preoperative CT was performed. The CT scanners and settings were Siemens Somatom Plus 4 Volume Zoom (140 kV, 159 A, 1 mm slice thickness), Siemens Somatom Emotion 6 (130 kV, 70 mA, 2 mm slice thickness), Toshiba Aquilion 4 slice (120 kV, 70 mA, 2 mm slice thickness), and Phillips Brilliance 180P2, 10 slice (120 kV, 30 mA, 1 mm slice thickness).

### Computed tomography image analysis

**Fig 1:**
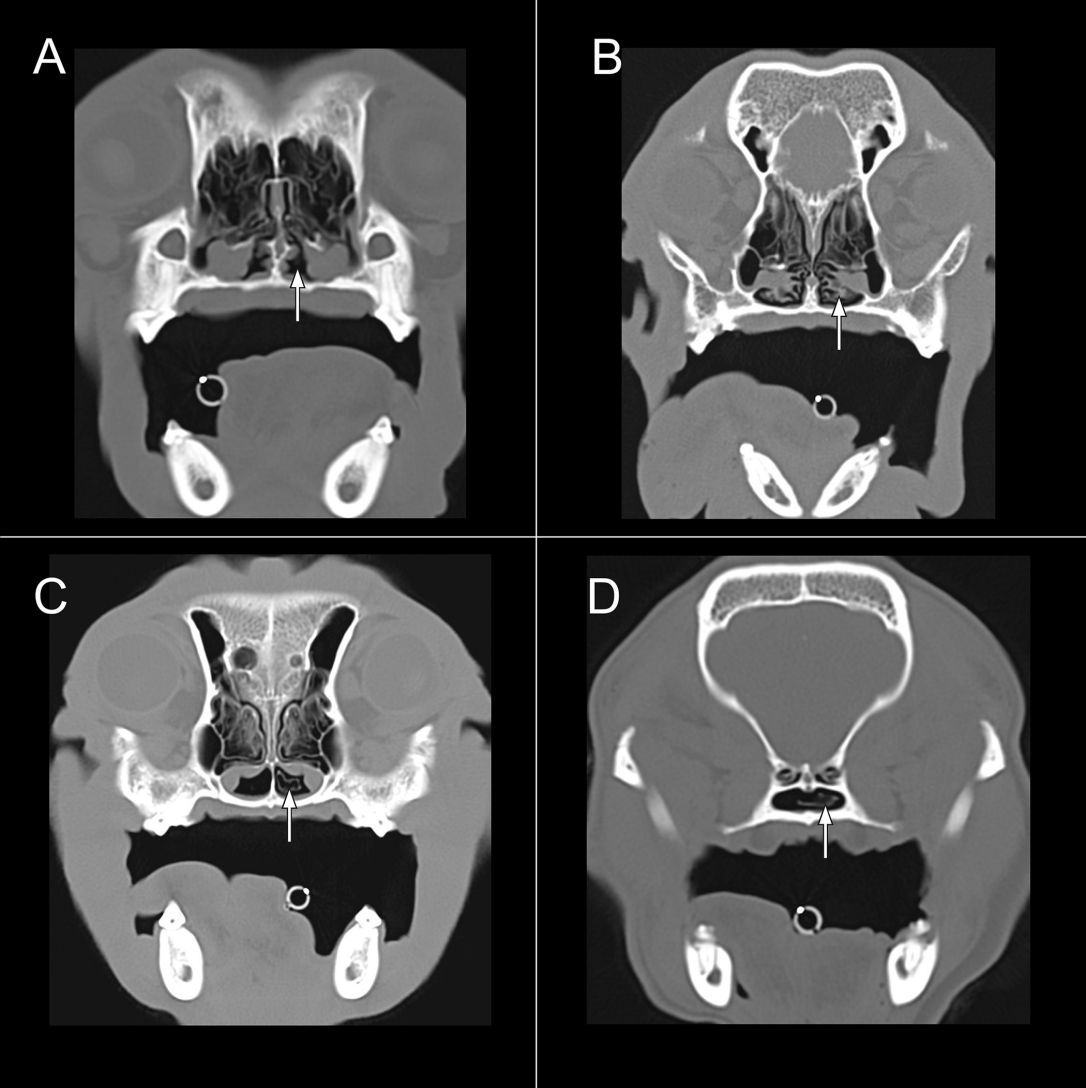
Grading system caudal aberrant turbinates (CAT). (A) Grade 0, arrow pointing at ventral nasal meatus void of turbinates. (B) Grade 1, arrow pointing at ventral nasal meatus with turbinates.(C) Grade 2, arrow pointing at turbinate in choana. (D) Grade 3, arrow pointing at turbinate in the bony nasopharyngeal meatus.

The grading system by Vilaplana Grosso et al, 2015, for assessing the presence and severity of caudal aberrant turbinates (CAT) was modified to four instead of five grades [64]. Grade 0 was allocated to cases with no CAT. Grade 1 characterized turbinates present in the ventral nasal meatus, grade 2 turbinates in the choanae and grade 3 turbinates in the bony part of the nasopharyngeal meatus. No difference in grading was made for unilateral or bilateral appearance (Fig 1).

**Fig 2:**
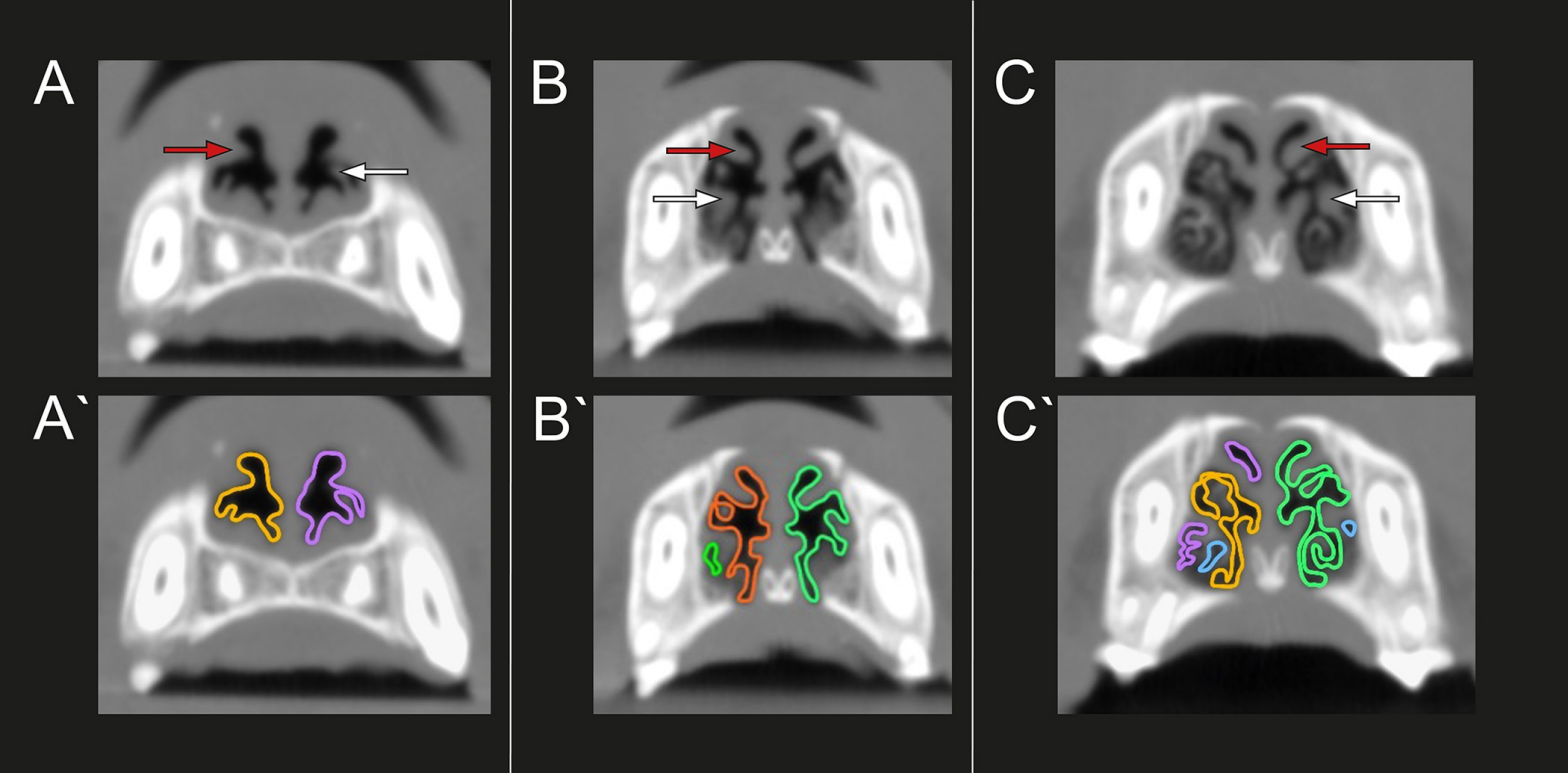
Locations A-C for the preoperative and postoperative CT-based measurements. Location A, (A) before, and (A’) after the measurement. Location B, (B) before, and (B’) after the measurement. Location C, (C) before, and (C’) after the measurement. Red arrows: dorsal turbinate, white arrows: ventral turbinate.

**Fig 3:**
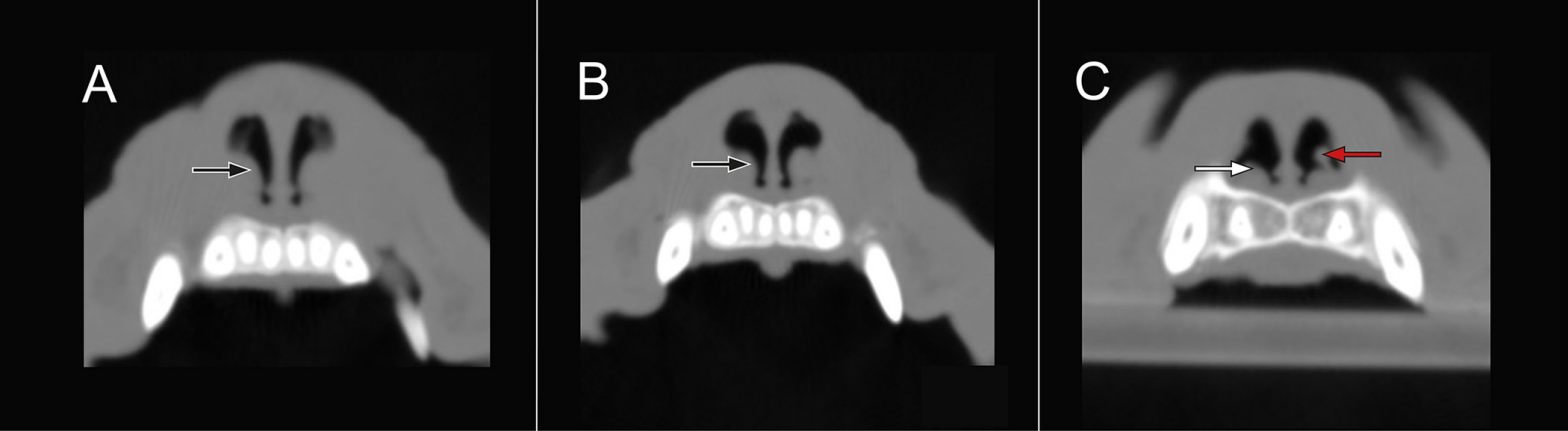
Nasal vestibule, rostral to the CT-scan-based measurements. (A-B) consecutive slices through the nasal vestibulum from rostral to caudal after resection of the vestibular fold, six months post-op. Black arrow: former attachment site of the vestibular fold. White arrow: nasal floor. Red arrow: the ventral turbinate’s head.

To calculate the effect of the RFVTR on the turbinates based on the pre- and postoperative CT images, a standardized method using DICOM imaging software (Horos v3.3.6) was used with the following settings for bone window measurements (WL: 300–600 HU, WW: 1500–2000 HU) and for soft tissue analysis (WL: 30–50 HU, WW: 300–600 HU). The settings were adjusted, based on the specific analysis and the CT scanner used. The pencil tool was used to calculate the surface area occupied by air in the rostral nasal chamber. To improve precision of the comparison between the pre- and postoperative CT, the rostral nose caudal to the vestibule was divided into three locations. Location A is situated at the ventral turbinate’s head, location B where the branching of the ventral turbinate starts, and location C is where the ventral turbinate showed maximum branching with still measurable airspaces detectable in between (Fig 2). The measurements were carried out caudally to the vestibular fold to avoid interference with the effect of the anatomical changes caused by the ala vestibuloplasty (Fig 3).

### Rhinoscopy

**Fig 4:**
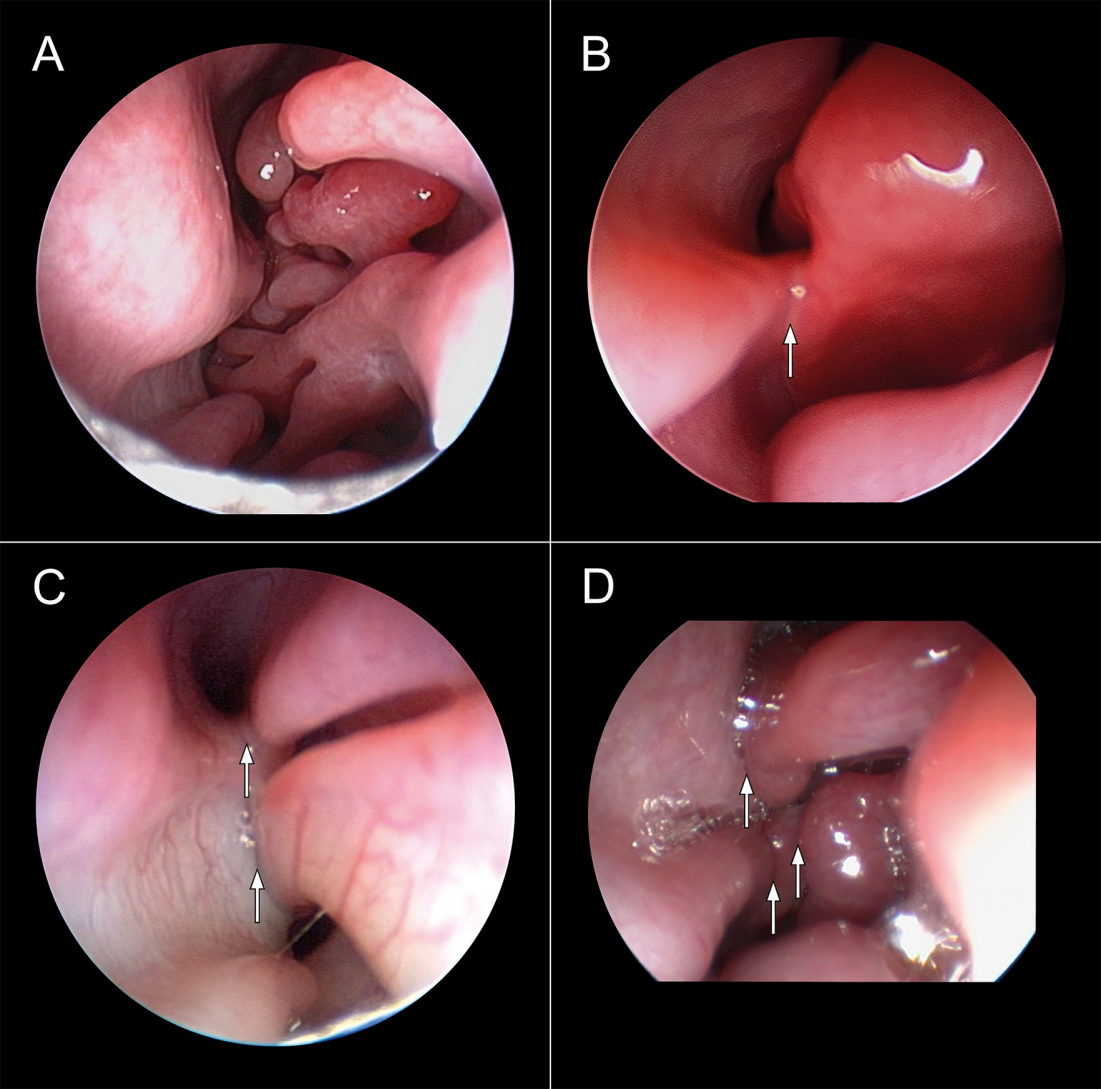
Grading system mucosal contact points. (A) Grade 0: no mucosal contact points. (B) Grade 1: one mucosal contact point (arrow). (C) Grade 2: two mucosal contact points (arrows). (D) Grade 3: three mucosal contact points (arrows).

A rigid 0-degree endoscope, 2.7 mm in diameter, 18 cm in length was used for anterograde rhinoscopy, and a 120-degree endoscope, 4 mm in diameter, 18 cm in length was used for retrograde rhinoscopy. During anterograde rhinoscopy the nasal vestibule, the nasal septum, the dorsal and ventral turbinates as well as the dorsal, the middle, the ventral and the common nasal meatuses were examined. Special emphasis was placed on the presence of hypertrophy of the dorsal and ventral turbinate and the amount of mucosal contact points (MCPs). The degree of intranasal obstruction was assessed by a scoring system based on the amount of MCPs present. Score 0 represented no visible contact points, score 1 represented one visible contact point, score 2 represented two contact points, and score 3 represented three or more contact points (Fig 4 and S1 Video). Retrograde rhinoscopy examined the overall width of the nasopharyngeal meatus and the presence of turbinates or pathological changes in the choanae or nasopharyngeal meatus.

### Radiofrequency volumetric turbinate reduction

Hypertrophic turbinates were reduced in volume using a bipolar radiofrequency probe (Reflex Ultra PTR, Smith &Nephew) together with the Coblator II radiofrequency unit under direct endoscopic visualization (Smith&Nephew, London, United Kingdom). The RF probe was introduced into each hypertrophic segment of a turbinate, following a rostro-caudal trajectory, with the objective of maintaining distance from the mucous membrane and the underlying bone. Upon achieving the intended position of the probe, the radiofrequency current was initiated at a power level of 2 watts, followed by gradual retraction of the probe. Meticulous care was taken to prevent pallid discoloration of the mucous membranes, as previously described [48]. If pale discoloration occurred retraction speed was adjusted accordingly.

### Additional multi-level surgery procedures

Additional MLS procedures consisted of palatoplasty, ala vestibuloplasty, sacculectomy, and unilateral cricoarytenoid lateralisation combined with arytenoid laryngoplasty in selected cases. Dogs with grade 3 laryngeal collapse that showed persistent dyspnea during recovery, were re-anesthetized and cricoarytenoid lateralisation combined with arytenoid laryngoplasty was performed [65]. The dogs were closely monitored during recovery and reunited with their owners after the endotracheal tube was removed. All patients were discharged on the same day.

### Follow-up

Follow-up assessments were conducted utilizing a standardized questionnaire at weeks 4, 24, 48, 72, 96 and 120, either through written responses or telephone interviews and during re-evaluation appointments after 24 weeks. At the 6-month postoperative re-examination, a CT of the head, anterograde and retrograde rhinoscopy, and endoscopy of the oral cavity were carried out.

### Statistics

Statistical analysis was performed with SPSS (IBM1 SPSS1 Statistics, version 28, 64-bit-version, IBM, Chicago, Ill). Shapiro-Wilk test was used to test for normality. All data was presented with a median (range) and non-parametrical tests were used to compare groups. A descriptive analysis of the data was performed and presented. The Friedman test was performed as a non-parametric statistical test to detect differences in rankings across multiple related groups or conditions. Subsequently, a bilateral (two-sided) post hoc analysis was conducted to identify specific pairs of groups demonstrating statistically significant differences. For post hoc analysis Wilcoxon signed-rank tests with Bonferroni correction were performed. A p-value of < 0.05 was considered significant.

## Results

### Study population and clinical findings

132 dogs met the inclusion criteria, and for 25% (33/132 dogs) 6-month postoperative imaging was available. The median follow-up was 58 weeks (range, 4–120 weeks). Dog breeds included French Bulldogs 73.5% (97/132), pugs 17.4% (23/132), English Bulldogs 4.5% (6/132), Boston Terriers 2.3% (3/132), Old English Bulldogs 1.5% (2/132), and one Boxer (0.8%). Male dogs were overrepresented (65.9%, 87/132).

The median age of the patient population at the time of surgery was 3.4 years (range 0.9–10 years). Old English Bulldogs had the youngest median age of 2 years (range, 1.5–3 years), followed by French Bulldogs’ median age 2.9 years (range, 0.9–10 years), English Bulldogs’ median age 3.5 years (range, 1.5–7 years), and pugs’ median age 5.2 years (range, 1.5–8 years). Overweight was present in 48.2% (41/85) of the dogs, including slight overweight in 22.3% (19/85), moderate overweight in 15.3% (13/85), and severe overweight in 10.6% (9/85). Prior upper airway surgery had been performed in 18.9% (25/132) with correction of stenotic nares in combination with palatoplasty (16/24), resection of laryngeal saccules (3/25), tonsillectomy (2/25) and LATE (1/25).

**Fig 5:**
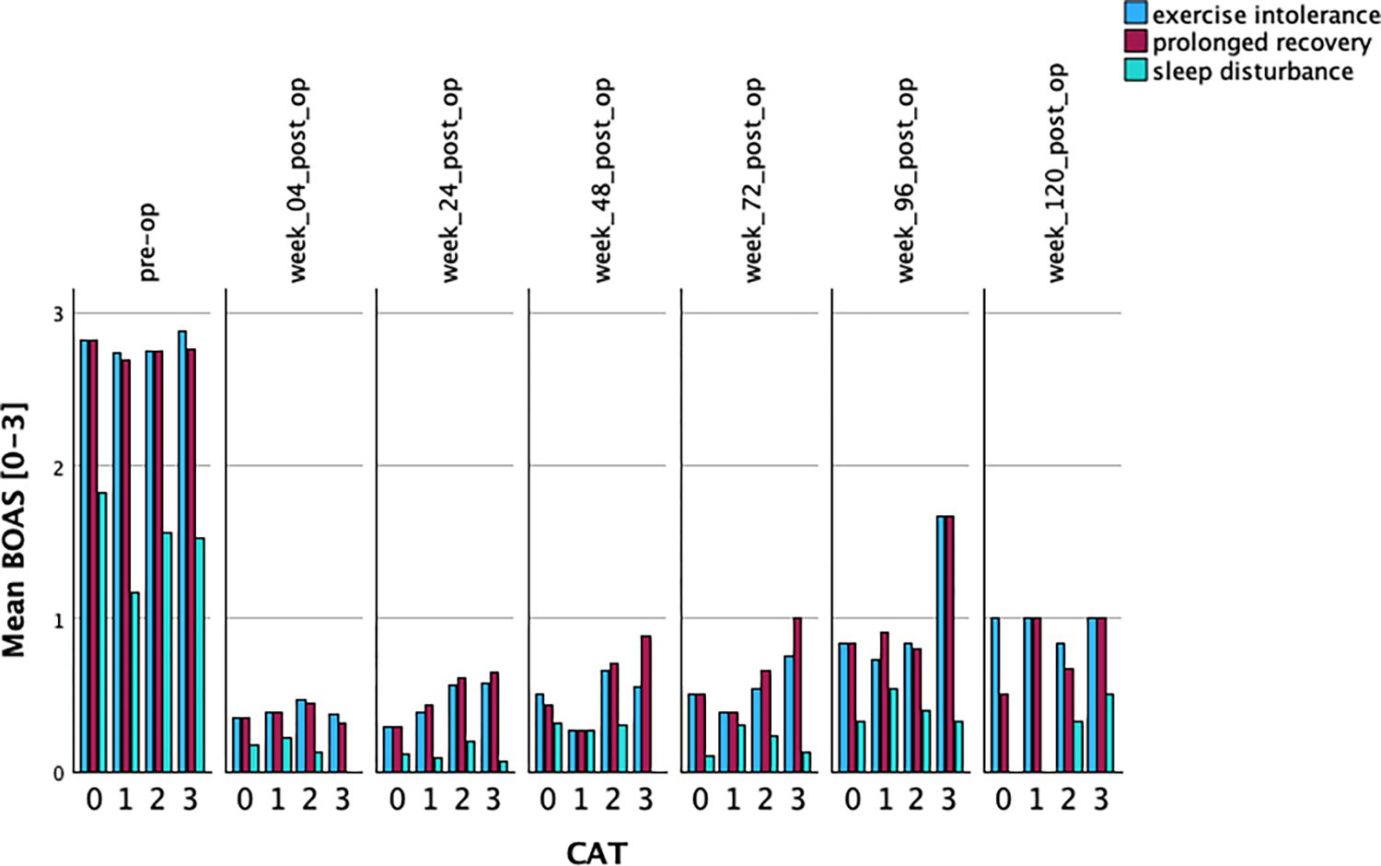
Changes in mean BOAS scores and the effect of CAT. Preoperative (pre-op), and postoperative (post-op) for weeks 4, 24, 48, 72, 96, and 120.

Most of the dogs (98.5%) were not able to maintain a normal body temperature during exercise or excitement. BOAS grading consisted of Grade 0 in 0.8% (1/132), grade 1 in 0% (0/132), grade 2 in 19.7% (26/132), and grade 3 in 79.5% (105/132) (Table 1 and S1 Table). More than half of the dogs (55.3%) showed clinical signs of sleep disturbance. Laryngeal collapse was diagnosed in 40.16% (51/127) with grade 2 in 26.8% (34/127) and grade 3 in 3.15% (4/127). All grade 3 dogs were older than 5 years (Fig 5).

**Table 1:** Friedmann test with bonferroni correction for pairwise comparison of the follow-up time points and the clinical parameters.

**overall**			
**comparisons follow-up**	**exercise intolerance [n = 590]**	**prolonged recovery [n = 589]**	**sleep disturbance [n = 587]**
**Friedmann Test**			
	<0.001	<0.001	<0.001
pairwise comparisons**			
pre-op—4 weeks	<0.001	<0.001	<0.001
pre-op—24 weeks	<0.001	<0.001	<0.001
pre-op—48 weeks	<0.001	<0.001	<0.001
pre-op—72 weeks	<0.001	<0.001	<0.001
pre-op—96 weeks	<0.001	<0.001	<0.001
pre-op—120 weeks	0.001	<0.001	0.006
all other time points	ns	ns	ns
**CAT 0**			
**comparisons follow-up**	**exercise intolerance [n = 85]**	**prolonged recovery [n = 85]**	**sleep disturbance [n = 85]**
**Friedmann Test**			
	<0.001	<0.001	<0.001
pairwise comparisons**			
pre-op—4 weeks	<0.001	<0.001	<0.001
pre-op—24 weeks	<0.001	<0.001	<0.001
pre-op—48 weeks	<0.001	0.003	<0.001
pre-op—72 weeks	<0.001	0.001	<0.001
pre-op—96 weeks	0.144	0.222	<0.001
pre-op—120 weeks	1	0.304	0.006
all other time points	ns	ns	ns
**CAT 1**			
**comparisons follow-up**	**exercise intolerance [n = 109]**	**Prolonged recovery [n = 109]**	**sleep disturbance [n = 109]**
**Friedmann Test**			
	<0.001	<0.001	0.003
pairwise comparisons**			
pre-op—4 weeks	<0.001	0.026	0.026
pre-op—24 weeks	<0.001	<0.001	0.001
pre-op—48 weeks	<0.001	<0.001	0.206
pre-op—72 weeks	<0.001	<0.001	0.522
pre-op—96 weeks	0.002	0.01	1
pre-op—120 weeks	1	1	1
all other time points	ns	ns	ns
**CAT 2**			
**comparisons follow-up**	**exercise intolerance [n = 326]**	**Prolonged recovery [n = 325]**	**sleep disturbance [n = 324]**
**Friedmann Test**			
	<0.001	<0.001	<0.001
pairwise comparisons**			
pre-op—4 weeks	<0.001	<0.001	<0.001
pre-op—24 weeks	<0.001	<0.001	<0.001
pre-op—48 weeks	<0.001	<0.001	<0.001
pre-op—72 weeks	<0.001	<0.001	<0.001
pre-op—96 weeks	<0.001	<0.001	<0.001
pre-op—120 weeks	0.011	0.001	0.235
all other time points	ns	ns	ns
**CAT 3**			
**comparisons follow-up**	**exercise intolerance [n = 70]**	**prolonged recovery [n = 70]**	**sleep disturbance [n = 69]**
**Friedmann Test**			
	<0.001	<0.001	<0.001
pairwise comparisons**			
pre-op—4 weeks	<0.001	<0.001	<0.001
pre-op—24 weeks	<0.001	<0.001	<0.001
pre-op—48 weeks	<0.001	0.004	0.001
pre-op—72 weeks	0.002	0.015	0.012
pre-op—96 weeks	1	1	1
pre-op—120 weeks	1	1	1
all other time points	ns	ns	ns

*Significance (2-sided), ns = not significant (p<0.05)

**Significance adjusted by the Bonferroni correction for multiple tests (degrees of freedom = 6)

### Diagnostic imaging and surgical complications

MCPs were present in all patients with excessive obliteration of the nasal meatuses (score 3) in 75.8% (100/132). CAT were present in most patients (87.1%, 115/132) with extension into the choanae (grade 2) in 56.8% (75/132), and into the nasopharynx (grade 3) in 12.9% (17/132). Two patients needed cricoarytenoid lateralisation in combination with arytenoid laryngoplasty due to a grade 3 laryngeal collapse.

No complications were encountered during surgery or recovery, and all dogs were discharged on the same day (n = 132). Post-operative complications associated with RFVTR, and overall complications were mild and consisted of serous nasal discharge in all dogs during the first week and signs of mild nasal congestion after 3–8 weeks in 24.2% (32/132) of the patients.

### Follow-up

**Fig 6:**
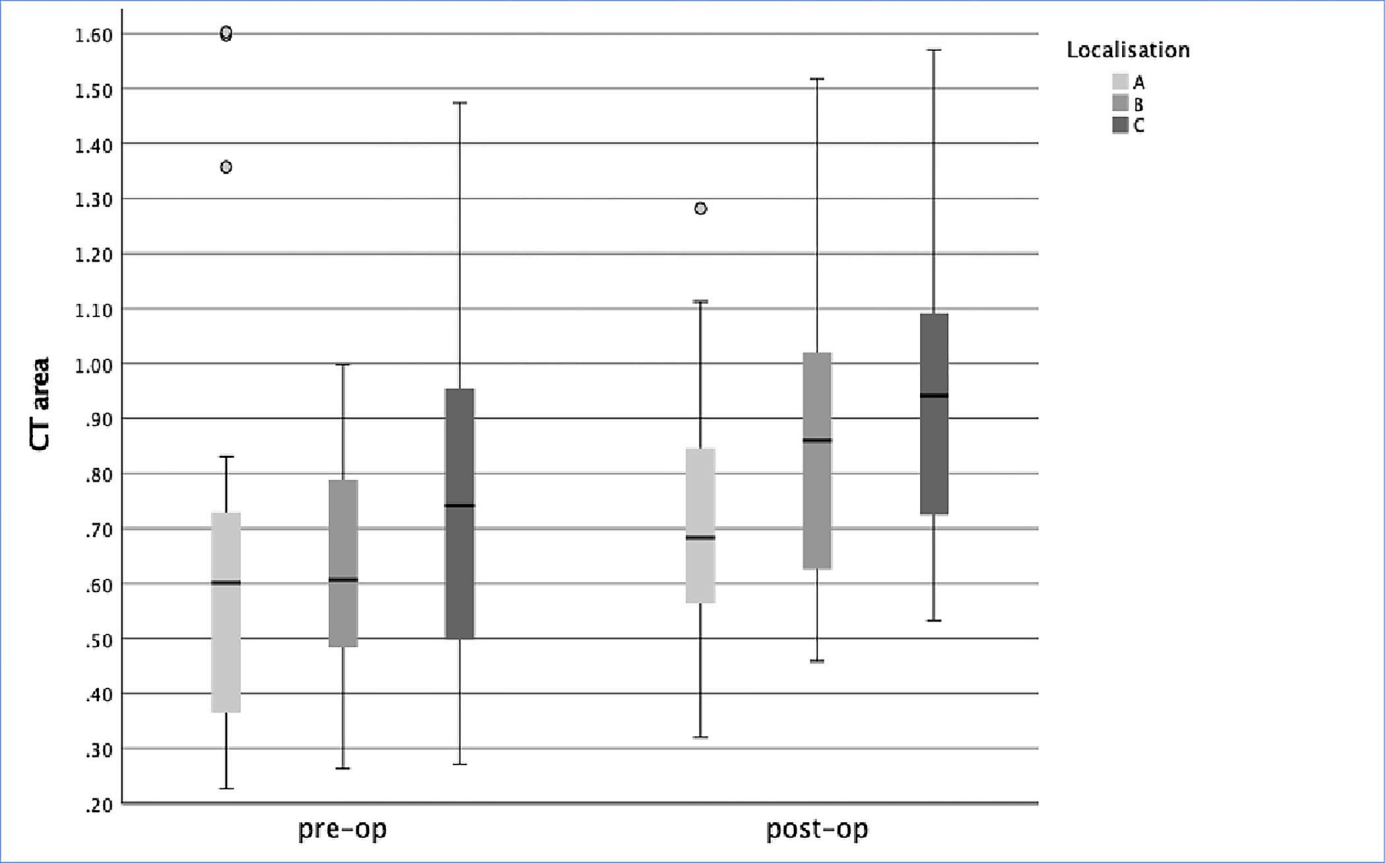
CT-based measurements of the rostral nose for locations A-C Preoperative measurements (pre-op) and postoperative measurements (post-op).

All clinical parameters for BOAS improved significantly for each time point of the follow-up in comparison to the pre-op results (Fig 5). However, no significant differences were observed when comparing the post-operative time points alone (p> 0.05, Table 1 and S1 Table). When the different follow-up points were compared with the preoperative findings and paired with the scores for CAT no significant difference for score CAT 0–3 was detected after week 72. After 72 weeks improvement was still present but not significant, especially for the parameters sleep disturbance in combination with CAT 2 and CAT 3. Exercise intolerance and prolonged recovery remained significantly improved in patients with CAT 2 over the whole follow-up period (Table 1 and Fig 5). A significant increase in the areas devoid of nasal structures within the rostral third of the nose was observed in the pre- and post-treatment CT measurements at location B (pre-op: 0.63 ± 0.27 cm^2^; post-op: 0.85 ± 0.28 cm^2^, p = <0.001) and location C (pre-op: 0.75 ± 0.31 cm^2^; post-op: 0.94 ± 0.27 cm^2^, p<0.001). No significant difference was found at location A in the rostral nose (pre-op: 0.68 ± 0.43 cm^2^; post-op: 0.70 ± 0.22 cm^2^, p = 0.106) (Table 2 and Fig 6). Intranasal obstruction was significantly less present after six months. In 45.5% (15/33) no MCPs were detected and grade 3 was not found in any of the patients (S2 Video).

**Table 2:** CT-based measurements of the rostral nose for locations A-C. * indicates significance between preoperative and postoperative measurements.

Area		Pre-op-Area [cm^2]	Post-op-Area [cm^2]	Wilcoxon Signed Rank Test [for median of differences between Pre- & Post-op-Areas]	
**A**	Mean	0.68	0.70	Total N	33.00
	Median	0.61	0.68	Test Statistic	371.00
	SD	0.43	0.22	Standard Error	55.97
	Variance	0.19	0.05	Standardized Test Statistic	1.62
	Range	2.04	0.98	**Asymptotic Sig. (2-sided test)**	**0.106**
**B**	Mean	0.63	0.85	Total N	33.00
	Median	0.62	0.86	Test Statistic	513.00
	SD	0.27	0.28	Standard Error	55.97
	Variance	0.07	0.08	Standardized Test Statistic	4.15
	Range	1.40	1.06	**Asymptotic Sig. (2-sided test)**	< 0.001*
**C**	Mean	0.75	0.94	Total N	32.00
	Median	0.74	0.94	Test Statistic	470.00
	SD	0.31	0.27	Standard Error	53.48
	Variance	0.10	0.08	Standardized Test Statistic	3.85
	Range	1.20	1.04	**Asymptotic Sig. (2-sided test)**	< 0.001*

## Discussion

While MLS with alleviation of intranasal stenosis shows superior results in comparison to traditional MLS in dogs with BOAS, a minimally invasive turbinoplasty technique has not been described in veterinary patients so far [5, 8, 11, 12, 66]. Intranasal obstruction is diagnosed by the presence and amount of MCPs. MCPs are thought to origin from rostral and caudal aberrant turbinates [5, 11, 12, 14]. Although the presence of CAT was a constant finding in our patients in 69.7% (grade 2 and 3), neither the presence, nor grade of CAT had a significant impact on exercise intolerance or prolonged recovery (Fig 5). Similar results have been described for Bulldogs without BOAS [64]. Interestingly, patients with CAT 0 and CAT 3 had the same results for exercise intolerance and prolonged recovery. We assume that the small patient number may have contributed to the lack of significance in these patients. Both groups (CAC 0 and CAC 3) consisted of only 17 patients each, in comparison to 75 patients in the CAT 2 group. In our experience, and referring to the visual examples of CAT available in the literature, CAT are rarely hypertrophied and contribute to the formation of MCPs mainly by being contacted by enlarged nasal turbinates or maxilloturbinates [14, 34]. We side with the interpretation by Liu et al., 2019 and Ginn et al., 2008, that caudally extending branches serve as a compensation for the restricted intranasal space in brachycephalic dogs, without adding to intranasal obstruction [6, 8]. Consequently, we recommend considering the renaming of these structures as nasopharyngeal turbinates (NPTs). Our results show, that RFVTR increases airway patency without reducing the number of turbinates or changing their architecture. The RFVTR significantly reduced the volume of the nasal turbinates, as evidenced by the scoring of the MCPs and measurements taken at three different locations in the CT images, both pre- and postoperatively. The decrease in intranasal obstruction among the control patients corresponds to the increase in areas occupied by air measured at location B and C. The lack of significant difference for location A can be attributed to that specific nasal area. The maxilloturbinate’s head is located caudal to the alar fold and extends caudally until the beginning of the branching. The maxilloturbinate’s head is one of the biggest intranasal obstructors in human patients [19, 43]. In our patients little to no obstruction was observed at this location due to the relatively wide nasal vestibule. We also observed that the maxilloturbinate’s head tends to be very variable in size. Most of the intranasal obstruction was identified predominantly in location B and C, at the level of the branching of the maxilloturbinate. The pathogenesis of hypertrophic turbinates in dogs with BOAS is unclear. Stenotic nares have been identified as the main contributor to intranasal pressure increase and are thought to either cause or predispose to the stenotic sites situated more caudally [2]. Complete obstruction of one nostril in mice causes unilateral turbinal atrophy on the affected side leading to the interpretation that reduced airflow and increased intranasal pressure contribute to the development of hypertrophic turbinates while a complete absence of airflow and pressure variation causes the opposite effect [67]. Dogs eliminate surplus body heat by panting. The efficacy of body temperature regulation depends on nasal anatomy, mucosal surface area and flow dynamics [68]. Three panting patterns have been recognized in the dog: 1) inhale and exhale through the nose, 2) inhale through the nose, exhale through nose and mouth, and 3) inhale and exhale through nose and mouth. In spite of high airflow resistance, the nasal respiratory pathway remains part of the breathing cycle, even in heavy panting [27]. The lateral nasal gland responds to panting with an almost 10-fold excretion increase resembling sweat glands in larger mammals [69]. With the evaporation of the humidity on the mucosal surface, blood in the venous plexus is cooled. Most heat exchange takes place at the maxilloturbinate [68]. Partial or complete obliteration caused by MCPs changes nasal airflow and reduces air/mucosal contact, theoretically reducing cooling-capacity and prolonging post-exercise recovery time. A reduction in mucosal surface area and venous plexus is likely to diminish the cooling efficacy even more. After partial or complete turbinectomy or in empty nose syndrome, humans paradoxically report the subjective sensation of nasal obstruction [15, 70, 71]. Computational fluid dynamics (CFD) have shown a reduction of mucosal interaction after turbinectomy [15, 18]. Humans perceive nasal airflow through a temperature gradient, mediated by stimulation of trigeminal cool afferents [70, 72]. A reduction in mucosal surface and thus trigeminal cool afferents has been discussed as an explanation for the lack of patient satisfaction after turbinectomy [16]. These findings should be considered in the treatment of hypertrophic turbinates in BOAS. We observe hypertrophy of the nasal (dorsal) turbinate with complete obstruction of the dorsal meatus in our patients on a regular basis. The dorsal meatus serves as a fast lane to transport odor to the olfactory recess. We assume, that reinstallation of a dorsal meatus by turbinoplasty could contribute to improving olfaction in dogs with BOAS. Since correction of stenotic nares serves the same purpose, objective evaluation of that effect remains difficult.

Mild complications related to RVFTR were found, which are comparable to those described in human medicine. The serous discharge and mild nasal congestion observed during the early post-op period is attributed to the initial phase of healing and irritation of the nasal mucosa at the probe insertion sites. [47, 73, 74]. In human patients, recurrence of clinical signs after turbinoplasty has been described within two to five years postoperatively [19, 38, 41, 73, 75]. Oechtering and others confirmed the regenerative nature of canine turbinates [12, 34]. During our follow-up period, we did not see rapid re-engorgement of the treated turbinals, that required another treatment. Finding the patients in need of a repeated RFVTR procedure, without regular endoscopy controls is challenging. Obstructive nasal pathways caused by MCPs anecdotical were thought to elicit stertor. In our experience, neither the physiologically occurring periodical engorgement of the nasal mucosa during the nasal cycle nor the pathological changes by MCPs can be picked up acoustically during routine clinical examination. The most important clinical sign for recurrence of hypertrophic turbinates would be a decrease in cooling ability of the dog, and we suggest that owners should be instructed accordingly. We feel that RFVTR is a relatively minor procedure can be repeated easily if needed and we expect complication rates to be low. Based on what is known we suggest regular re-schedules including rhinoscopy every two years after surgery, and close monitoring of the dog’s ability to regulate core body temperature.

Most of the study participants exhibited a significant improvement in exercise tolerance, recovery time, sleep quality, and reduction of noisy breathing throughout the entire duration of the follow-up period. Without a control group to compare the effect of MLS with and without RFVTR these findings are of questionable value. We noticed slight decline in owner satisfaction at week 96. This can either be attributed to recurrence of BOAS after surgery or a habituation effect to the situation after surgery. The owners that were less satisfied with the procedure after 9 months were the same that were moderately satisfied or dissatisfied at week 18 and week 72. Furthermore, the number of owners still participating in the follow-up was reduced at week 96 and 120 (Table 1). This possibly overemphasizes the decrease in owner satisfaction after week 96. We consider our follow-up of 30 months to not be long enough to reflect the influence of aging and its effect on the soft tissues of the upper airways.

Our findings demonstrate that RFVTR is turbinate-sparing, minimal-invasive, and effective. It can be included into MLS without increasing post-operative morbidity or mortality. Our results show that hospitalization after RFVTR is not needed, which reduces risk factors associated with BOAS, out-of-hour work, and overall costs [20]. Since our BOAS dogs are discharged on the same day, we extensively engage in pre- and post-operative education to inform owners about the specific clinical signs they need to be vigilant about. This education empowered the owners to provide accurate and informed responses. As a result, it is hypothesized that the outcomes of the follow-up period closely reflect the actual clinical condition of the dogs.

While every effort was made to minimize confounding variables, it is essential to acknowledge several limitations. Due to the retrospective study design, not all parameters could be controlled, and the study lacks a control group. Though a reasonable number of cases were included, the overall sample size is small.

The reason for the relatively limited number of patient owners who chose for a complete clinical re-examination after six months remains unclear. It is worth noting that dog owners tend to express significant concerns regarding potential anesthetic complications and often seek to avoid general anesthesia unless clearly necessitated by a deterioration of BOAS. This circumstance may have introduced bias, potentially resulting in either an overrepresentation of less satisfied or more satisfied patient owners. However, we did not observe any indication of either negative or positive bias within the follow-up data. The use of questionnaires for gathering data on the clinical outcome of a group of patients is considered susceptible to misrepresentation and bias. [76].

In conclusion the RFVTR seems to be a minimally invasive, turbinate-sparing, feasible and effective technique to alleviate intranasal obstruction in dogs with brachycephalic obstructive airway syndrome. Further studies with focus on the actual histological effect of RFA on canine maxilloturbinals, including long-term controls with rhinoscopy, CT imaging and CFD are needed.

## Supporting information

S1 Video Hypertrophic turbinates. Rhinoscopy with grade 3 intranasal obstruction before surgery. Left nasal chamber.
(MP4)

S2 Video Turbinates without mucosal contact points (MCPs) after RFVTR. Rhinoscopy six months after RFVTR. Left nasal chamber of the same patient as in S1 Video.
(MP4)

S1 Table BOAS grades and clinical signs before and after MLS including RFVTR. The data is based on the clinical exams and the answers to the questionnaires.
(DOCX)
