# Supplementary material for: Retrospective evaluation of radiofrequency volumetric tissue reduction for hypertrophic turbinates in dogs with brachycephalic obstructive airway syndrome
Source: PLoS One. 2024 Jul 1;19(7):e0306391. doi: 10.1371/journal.pone.0306391 (PMC11216595; doi:10.1371/journal.pone.0306391)
Supplement: S1 Table — The data is based on the clinical exams and the answers to the questionnaires. (DOCX) [file pone.0306391.s003.docx]

| **time points** | **BOAS** | **exercise intolerance** | **prolonged recovery** | **sleep disturbance** | **owner satisfaction** |
| --- | --- | --- | --- | --- | --- |
|  | **grade** |  |  |  |  |
|  | **clinical exam** | **Descriptive Evaluation of Questionnaires** | | | |
| pre-op | 0 | 1 (0.8%) | 1 (0.8%) | 40 (30.3%) | - |
|  | 1 | 0 | 1 (0.8%) | 17 (12.9%) | - |
|  | 2 | 26 (19.7%) | 27 (20.5%) | 38 (28.8%) | - |
|  | 3 | 105 (79.5%) | 103 (78.0%) | 35 (26.5%) | - |
|  | total | 132 (100%) | 132 (100%) | 130 (98.5%9 | - |
| 4 weeks | 0 | 77 (59.7%) | 81 (62.8%) | 111 (86.7%) | 90 (69.8%) |
|  | 1 | 48 (37.2%) | 43 (33.4%) | 17 (13.3%) | 33 (25.6%) |
|  | 2 | 4 (3.1%) | 5 (3.9%) | 0 | 6 (4.7%) |
|  | 3 | 0 | 0 | 0 | 0 |
|  | total | 132 (100%) | 129 (97.7%) | 128 (97.0%) | 129 (97.7%) |
| 24 weeks | 0 | 66 (55.0%) | 65 (54.6% | 103 (85.9%) | 77 (64.7%) |
|  | 1 | 49 (40.8%) | 44 (37.0%) | 16 (13.3%) | 36 (30.3%) |
|  | 2 | 5 (4.2%) | 10 (8.4%) | 1 (0.8%) | 6 (5.0%) |
|  | 3 | 0 | 0 | 0 | 0 |
|  | total | 120 (90.9%) | 119 (90.2%) | 120 (90.9%) | 119 (90.2%) |
| 48 weeks | 0 | 46 (52.9%) | 43 (49.4%) | 66 (75.9%) | 58 (66.7%) |
|  | 1 | 34 (39.1%) | 36 (41.4%) | 19 (21.8%) | 22 (30.3%) |
|  | 2 | 7 (8.0%) | 8 (9.2%) | 2 (2.3%) | 7 (5.0%) |
|  | 3 | 0 | 0 | 0 | 0 |
|  | total | 87 (65.9%) | 87 (65.9%) | 87 (65.9%) | 87 (65.9%) |
| 72 weeks | 0 | 36 (54.5%) | 31 (41.1%) | 52 (78.8%) | 46 (69.7%) |
|  | 1 | 25 (39.7%) | 29 (43.9%) | 14 (21.2%) | 13 (19.7%) |
|  | 2 | 5 (7.6%) | 6 (9.0%) | 0 | 7 (10.6%) |
|  | 3 | 0 | 0 | 0 | 0 |
|  | total | 66 (50%) | 66 (50%) | 66 (50%) | 66 (50%) |
| 96 weeks | 0 | 14 (31.1%) | 13 (28.9%) | 28 (62.2%) | 27 (60.0%) |
|  | 1 | 23 (51.1%) | 24 (53.3%) | 15 (33.3%) | 10 (22.2%) |
|  | 2 | 8 (17.8%) | 8 (17.8%) | 2 (4.4%) | 7 (15.0%) |
|  | 3 | 0 | 0 | 0 | 1 (2.2%) |
|  | total | 45 (34.1%) | 45 (34.1%) | 45 (34.1%) | 45 (34.1%) |
| 120 weeks | 0 | 2 (18.8%) | 4 (36.4%) | 8 (72.2&) | 8 (72.7%) |
|  | 1 | 8 (72.2%) | 6 (54.5%) | 3 (27.2%) | 1 (9.1%) |
|  | 2 | 1 (9.6%) | 1 (9.1%) | 0 | 2 (18.2%) |
|  | 3 | 0 | 0 | 0 | 0 |
|  | total | 11 (8.3%) | 11 (8.3%) | 11 (8.3%) | 11 (8.3%) |
